# Midwall ejection fraction for assessing systolic performance of the hypertrophic left ventricle

**DOI:** 10.1186/1476-7120-10-45

**Published:** 2012-11-20

**Authors:** Hisao Yoshikawa, Makoto Suzuki, Go Hashimoto, Yukiko Kusunose, Takenori Otsuka, Masato Nakamura, Kaoru Sugi

**Affiliations:** 1 Division of Cardiovascular Medicine, Toho University Ohashi Medical Center, 2-17-6 Ohashi, Meguro-ku, Tokyo, Japan

**Keywords:** Midwall ejection fraction, Left ventricular systolic function, Left ventricular hypertrophy, Speckle tracking echocardiography

## Abstract

**Background:**

In patients with left ventricular hypertrophy (LVH), LV midwall fractional shortening (FS) is used as a measure of LV systolic performance that is more physiologically appropriate than conventional FS. For evaluation of LV volume and ejection fraction (EF), 2-dimensional (2D) echocardiography is more accurate than M-mode echocardiography. The purpose of this study was to assess systolic performance by midwall EF using 2D speckle tracking echocardiography (STE).

**Methods:**

Sixty patients were enrolled in the study. Patients were divided into two groups with LVH (n = 30) and without LVH (control group, n = 30). LV systolic function was compared between the two groups and the relationships of left ventricular mass index (LVMI) with LV systolic parameters, including midwall EF, were investigated.

**Results:**

Midwall EF in the LVH group was significantly lower than that in the control group (42.8±4.4% vs. 48.1±4.1%, p <0.0001). Midwall FS was also significantly lower in the LVH group (13.4±2.8% vs. 16.1±1.5%, p <0.0001), but EF did not differ significantly between the two groups. There were significant correlations between midwall EF and LVMI (r=0.731, p <0.0001) and between midwall FS and LVMI (r=0.693, p <0.0001), with midwall EF having the higher correlation.

**Conclusions:**

These results show that midwall EF can be determined using 2D STE. Midwall EF can be used to monitor LV systolic dysfunction, which is not possible with conventional EF. Evaluation of midwall EF may allow assessment of new parameters of LV systolic function in patients with LV geometric variability.

## Introduction

Left ventricular (LV) systolic performance is often assessed by ejection fraction (EF) and fractional shortening (FS). It is said that despite depression of LV systolic function, LVEF and LVFS are preserved in patients with left ventricular hypertrophy (LVH) [1-6]. However, assessment of LV systolic function at the endocardial surface is thought to reflect a geometric change of the LV, rather than the contractile function of the myocardium [4]. Midwall FS has been used as a more physiologically appropriate measurement of LV systolic performance in patients with LVH, compared to conventional FS, and decreased midwall FS is predictive of subsequent morbidity and mortality [1-9]. However, calculation of midwall FS is based on a limited region of the LV, rather than the whole left ventricle. Therefore, evaluation of midwall FS for assessment of LV systolic performance may not be applicable in patients with variable LV geometries. Two-dimensional (2D) echocardiography is more accurate for evaluating LV volume and EF compared with M-mode echocardiography, and 2D speckle tracking echocardiography (STE) allows measurement of LV volume and EF without manual tracking [10,11]. Therefore, the purpose of this study was to investigate the utility of midwall EF using 2D STE.

## Methods

### Population and study protocol

Sixty patients were enrolled in the study. Patients were divided into two groups with LVH (n = 30) and without LVH (control group, n = 30). LVH was defined as an LV mass index (LVMI) >96 g/m^2^ for women and >114 g/m^2^ for men. The causative of patients with LVH was essential hypertension with the exception of chronic renal failure, idiopathic hypertrophic cardiomyopathy, amyloidosis. Patients with myocardial infarction, atrial fibrillation, valvular disorders, and any other structural heart disease were excluded. Patients with poor echocardiographic image quality in the apical 2-chamber and 4-chamber views were also excluded. LV function was measured using conventional echocardiography, tissue Doppler imaging (TDI), and 2D-STE. LV systolic function was assessed by EF, midwall FS, midwall EF, and longitudinal strain. Systolic function was compared between the two groups and the relationships of LVMI with LV systolic parameters, including midwall EF, were investigated. Ethical review board approval from our hospital was obtained.

### Conventional echocardiography

Echocardiographic studies were performed using commercial equipment (X3 transducer, Philips iE33 system) with the patient in the left lateral decubitus position. Images were obtained using a 3.5-4.0 MHz transducer in the parasternal short-axis and apical 4-chamber views [12]. Interventricular septal thickness (IVST), posterior wall thickness (PWT), left ventricular end-diastolic diameter (LVDd), left ventricular end-systolic diameter (LVDs), left atrial dimension (LAD), stroke volume (SV), and left ventricular fractional shortening (LVFS) were determined using standard echocardiographic 2D or M-mode measurements. The IVST and PWT were measured in end diastole. LV mass was calculated from 2D echocardiographic measurements using the M-mode formula [13] and was normalized to body surface area. Mitral inflow velocity was traced and the peak early (E) and late (A) mitral flow velocities, the ratio of the early to late peak velocities (E/A), and the deceleration time (DCT) of the E velocity were derived from the velocity data. Midwall FS methods used the fact that the volume of myocardium between the midwall and the endocardium must be preserved although thickness of it was changed during cardiac cycle. Then, midwall FS was calculated using the model of Shimizu et al. [1,5-7], as follows:

1. *Midwall FS* = (*LVID*_*d*_ + *H*_*d*_/2) − (*LVID*_*s*_ + *H*_*s*_/2)/(*LVID*_*d*_ + *H*_*d*_/2).

2. Volume of myocardium between the midwall and the endocardium = (*LVID*_*d*_ + *H*_*d*_/2)^3^ − *LVID*_*d*_^3^ = (*LVID*_*s*_ + *H*_*s*_/2)^3^ − *LVID*_*s*_^3^.

3. *H*_*d*_ = *PWT* + *IVST.*

where LVID is the LV internal dimension, _d_ is end diastole, _s_ is end systole, and H is the shell thickness. In diastole, the inner and outer shells have, by definition, equal thickness given by (PWT + IVST).

### Tissue Doppler imaging

TDI was performed in all patients with images taken based on the guidelines of the American Society of Echocardiography [14]. Using the 4-chamber view, a 5-mm sample volume was placed at the septal and lateral border of the mitral annulus. Annular velocities were displayed in septal and lateral pulsed-wave TDI and the early systolic mitral annular velocity (S’), the early diastolic mitral annular velocity (E’), and the late diastolic annular velocity (A’) were determined from the average of septal and lateral data from the TDI recordings. The mitral E/E’ ratio was also calculated.

### Real-time 2D imaging

2D image analysis was performed on digitally stored images (X3 transducer, Philips iE33 system). Real-time 2D data sets were obtained from the apical 2-chamber and 4-chamber images. Images were recorded to allow for reliable operation of the software (Q-Lab, Version 7.0, Philips Healthcare).

### LV systolic strain measurement

Myocardial longitudinal strain measurement was assessed on 4-chamber image with speckle tracking analysis. The traced endcardium is automatically divided into six segments; septal, anteroseptal, anterior, lateral, posterior, and inferior. The average peak strain measured in the longitudinal directions defined as the longitudinal strain.

### Midwall EF measurement

**Figure 1:**
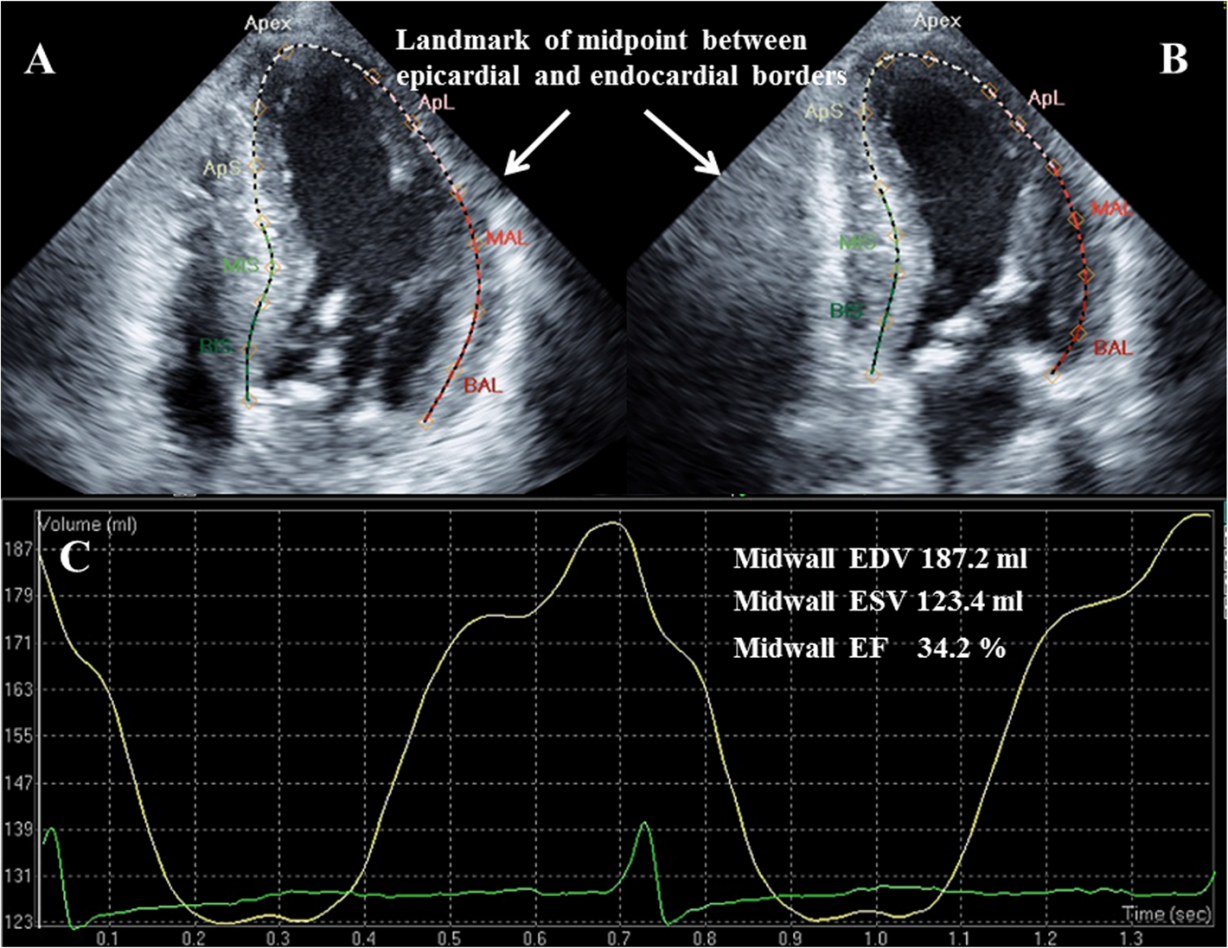
**Examples of midwall EF measurements.** (**A**) 4-chamber view. (**B**) 2-chamber view. The positioning of the midwall is determined by the landmark of the midpoint between the epicardial and endocardial borders. (**C**) Examples of midwall volume curve using speckle tracking method. EF = ejection fraction. EDV = end diastolic volume; ESV = end systolic volume.

The midwall EF measurements were obtained using semiautomated speckle tracking (Q-Lab, Version 7.0, Philips Healthcare). Image acquisition was performed with end-expiratory breath holding to reduce the scattering of values. One cardiac cycle was analyzed in each patient. The position of the midwall was determined using the landmark of the midpoint between the epicardial and endocardial borders depending on the LV wall thickness. Anatomic landmarks, including these midpoints and a point on the apical endocardium, were manually initialized at the end of diastole only. Following this initialization, the software automatically positioned sixteen regions of interests (ROI) on the midwall LV cavity surface. In systole, the initial ROI was not positioned at the midpoint of the wall thickness because systolic thickening of the inner layer is larger than that of the outer layer. Further manual adjustments of the position of the ROI in the end-diastolic frame were performed as necessary [10]. A representative case is shown in Figure 1A and 1B. Then, we automatically obtained the volume curve using a speckle tracking algorithm throughout the cardiac cycle and measured the end diastolic and end systolic volumes. A volume curve obtained with the speckle tracking algorithm in a representative case is shown in Figure 1C. Midwall EF was then calculated by the biplane method using the average value in the apical 4-chamber and 2-chamber views.

### EF measurement

These data were obtained using the same method as that for midwall EF measurement, except for the positions of ROIs, which were based on anatomic landmarks, including septal and lateral points on the mitral annulus and a point on the apical endocardium. The EF was then calculated from apical 4-chamber and 2-chamber views, as for the midwall EF in 2D STE.

### Statistical analysis

Data are expressed as means ± SD. A Student *t*-test was used to compare continuous variables and a χ^2^ test was used for categorical variables. Simple linear regression analysis was used to evaluate relationships between variables of interest, with p *<* 0.05 considered to indicate significance.

## Results

### Patient characteristics

Of the 69 patients who were initially recruited, 9 were excluded because the echocardiographic image quality was unsuitable for quantitative 2D STE analysis. Thus, 60 patients were subsequently enrolled in the study. Patients were divided into two groups with LVH (n = 30) and without LVH (control group, n = 30). The characteristics of the patients in these groups are shown in Table 1. There were no significant differences in mean age, gender, height, weight, body mass index, and systolic and diastolic blood pressure between the two groups. Frequencies of diabetes mellitus and hyperlipidemia did not differ significantly between the groups, but the frequency of hypertension in the LVH group was significantly higher than that in the control group.

**Table 1:** Patient characteristics in the LVH and control groups

**Item**	**LVH (n=30)**	**Control (n=30)**	**P-value**
Age (years)	66.0±15.7	62.9±14.5	0.4321
Male (%)	18 (60%)	17 (56%)	0.5893
Height (cm)	160.6±9.2	161.6±9.6	0.6551
Weight (kg)	58.9±11.6	59.2±11.3	0.9121
BMI (kg/m^2^)	22.7±3.4	22.5±2.7	0.7824
Systolic blood pressure (mmHg)	133.3±13.4	122.7±12.3	0.1012
Diastolic blood pressure (mmHg)	75.6±10.9	69.4±10.4	0.0516
Hypertension	30 (100%)	5 (16%)	<0.0001
Diabetes mellitus	5 (16%)	4 (13%)	0.5892
Hyperlipidemia	12 (40%)	8 (27%)	0.1257

Data are shown as a number (%) or mean ± S.D. LVH = left ventricular hypertrophy; BMI = body mass index. There were no significant differences in mean age, gender, height, weight, BMI, systolic and diastolic blood pressure between the two groups. Frequencies of diabetes mellitus and hyperlipidemia did not differ significantly between the two groups, but the frequency of hypertension in the LVH group was significantly higher than that in the control group.

### Echo parameters in conventional echocardiography

Echo parameters in the LVH and control groups are shown in Table 2. The IVST and PWT in the LVH group were larger (11.9±3.2 mm vs. 9.8±1.1 mm, p <0.0001; 12.7±2.1 mm vs. 9.9±1.2 mm, p <0.0001, respectively) and the LVMI was higher (132.2±28.3 g/m^2^ vs. 86.9±10.8 g/m^2^, p <0.0001) compared to the respective values in the control group. LVDd, LVDs, LVFS, E velocity, A velocity, and E/A did not differ between the two groups.

**Table 2:** Echo parameters in conventional echocardiography in the LVH and control groups

**Item**	**LVH (n=30)**	**Control (n=30)**	**P-value**
HR (bpm)	67.7±9.4	67.9±10.5	0.9252
LAD (mm)	40.6±4.7	35.6±3.6	0.0023
IVST (mm)	11.9±3.2	9.8±1.1	<0.0001
PWT (mm)	12.7±2.1	9.9±1.2	<0.0001
LVDd (mm)	43.2±4.9	43.1±4.1	0.9453
LVDs (mm)	26.5±3.9	26.9±3.4	0.6836
SV (mL)	56.1±15.7	55.2±13.8	0.8983
LVFS (%)	38.8±4.9	37.9±3.5	0.3844
LVMI (g/m^2^)	132.2±28.3	86.9±10.8	<0.0001
DCT (ms)	272.9±60.1	249.8±71.8	0.1823
E velocity (m/s)	56.7±14.8	61.8±16.1	0.1995
A velocity (m/s)	76.6±25.9	71.4±19.6	0.3847
E/A	0.81±0.33	0.97±0.52	0.1784

Data are shown as a number (%) or mean ± S.D. HR = heart rate; LAD = left atrial dimension; IVST = interventricular septal thickness; PWT = posterior wall thickness; LVDd = left ventricular end-diastolic diameter; LVDs = left ventricular end-systolic diameter; SV = stroke volume; LVFS = left ventricular fractional shortening; LVMI = left ventricular mass index; DCT = deceleration time of the E-wave; E velocity = peak early mitral flow velocity; A velocity = peak late mitral flow velocity; E/A = ratio of mitral E and A. IVST and PWT in the LVH group were longer and LVMI was higher compared to the respective values in the control group. LVDd, LVDs, LVFS, E velocity, A velocity, E/A did not differ between the two groups.

### Systolic echo and TDI parameters

Systolic echo and TDI parameters are shown in Table 3. The midwall EF in the LVH group was significantly lower than that in the control group (42.8±4.4% vs. 48.1±4.1%, p <0.0001). Midwall FS and longitudinal strain were also both significantly lower in the LVH group (13.4±2.8% vs. 16.1±1.5%, p <0.0001; -12.7±2.8% vs. -15.1±2.2%, p =0.0006, respectively), but EF did not differ significantly between the two groups (58.7±4.8% vs. 59.3±5.5%, p =0.6496). S’ and E’ in the LVH group were both significantly lower than those in the control group (7.6±1.1 cm/s vs. 9.0±1.6 cm/s, p =0.0021; 6.7±2.1 cm/s vs. 8.7±2.4cm/s, p =0.0013, respectively), and E/E’ was higher significantly in the LVH group (9.0±3.1 cm/s vs. 7.4±2.1 cm/s, p =0.0148).

**Table 3:** Systolic echo parameters in the LVH and control groups

**Item**	**LVH (n=30)**	**Control (n=30)**	**P-value**
EF (%)	58.7±4.8	59.3±5.5	0.6496
Midwall EF (%)	42.8±4.4	48.1±4.1	<0.0001
Midwall FS (%)	13.4±2.8	16.1±1.5	<0.0001
Longitudinal strain (%)	−12.7±2.8	−15.1±2.2	0.0006
S’ (cm/s)	7.6±1.1	9.0±1.6	0.0021
A’ (cm/s)	8.7±1.7	9.4±1.9	0.1749
E’ (cm/s)	6.7±2.1	8.7±2.4	0.0013
E/E’	9.0±3.1	7.4±2.1	0.0148

Data are shown as a number (%) or mean ± S.D. TDI = tissue Doppler imaging; EF = ejection fraction; midwall EF = midwall ejection fraction; midwall FS = midwall fractional shortening; S’ = peak systolic annular velocity; E’ = early diastolic mitral annular velocity; A’ = late diastolic mitral annulus velocity; E/E’ = ratio of E to E’. Midwall EF in the LVH group was significantly lower than that in the control group. Midwall FS and longitudinal strain were also significantly lower in the LVH group, but EF did not differ significantly between the two groups. S’ and E’ in the LVH group were lower than in the control group. E/E’ was higher significantly in the LVH group.

### Relationships between echo parameters and LVMI

**Figure 2:**
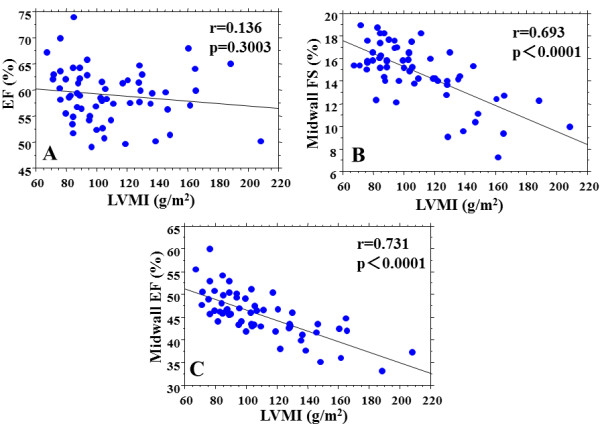
**Relationships between LVMI and systolic parameters in the 60 subjects in the study.** (**A**) Relationship between LVMI and EF. (**B**) Relationship between LVMI and midwall FS. (**C**) Relationship between LVMI and midwall EF. The abbreviations are the same as those in Table 3. LVMI = left ventricular mass index.

Relationships between echo parameters and LVMI are shown in Table 4 and Figure 2. LVMI was not correlated with EF (r=0.136, p=0.3003) (Figure 2A), but was significantly correlated with midwall FS (r=0.693, p<0.0001) (Figure 2B) and midwall EF (r=0.731, p<0.0001) (Figure 2C). LVMI was correlated with longitudinal strain (r=0.552, p<0.0001). LVMI was also correlated with S’ (r=0.386, p=0.0023), E’ (r=0.389, p=0.0021), and E/E’ (r=0.292, p=0.0234). Midwall EF showed the highest correlation with LVMI.

**Table 4:** Relationships between LVMI and echo parameters

**Factor**	**Correlation coefficient (r)**	**P-value**
EF	0.136	0.3003
Midwall EF	0.731	<0.0001
Midwall FS	0.693	<0.0001
Longitudinal strain	0.552	<0.0001
S’	0.386	0.0023
A’	0.176	0.1794
E’	0.389	0.0021
E/E’	0.292	0.0234

The abbreviations are the same as those in Table 3. LVMI = left ventricular mass index; The number of samples is 60 in this study. EF did not correlate with LVMI. There were significant correlations between LVMI and midwall EF, between LVMI and midwall FS. There was also significant correlatioms between LVMI and longitudinal strain. S’ and E’ also correlated with LVMI. Midwall EF had the highest correlation with LVMI.

### Reproducibility

The reproducibility of the EF and midwall EF measurements was evaluated by calculating the intra- and interobserver variability in 30 of the 60 patients selected at random. The intra- and interobserver variabilities were 4.1±3.3% and 4.8±3.2%, respectively, for EF, and 6.1±4.4% and 6.4±4.2%, respectively, for midwall EF.

## Discussion

The midwall EF in the LVH group was significantly lower than that in the control group and midwall EF was correlated with the degree of LVH. Our study showed the utility of midwall EF for assessing systolic performance of the hypertrophic left ventricle. Also, this method may be clinically useful and is likely to have low observer variability. As far as we are aware, there has only been one previous study of midwall EF. Jung et al. found that the midwall EF also discriminates the systolic function between patients with LVH and normal subjects [4], but the method used required manual tracing of echocardiographic images and a complicated calculation of echocardiographic data, which limits the clinical utility.

LVH is an independent predictor of adverse cardiovascular events in hypertension [9,15]. Accurate assessment of cardiac function in patients with LVH is important in clinical practice. LV systolic function has been wildly assessed as the ratio of observed LV endocardial FS or EF to value predicted by the level of end-systolic stress in normal subjects [3]. The degree of shortening and the level of opposing forces in myocardium in patients with LVH is different from normal subjects [6]. The previous study reported that LVEF and LVFS are preserved in patients with LVH, despite depression of LV myocardial systolic function [1,3-6]. In this study, the midwall EF in the LVH group was significantly lower than that in the control group and the correlation of LVMI with midwall EF was higher than that with any other parameters, including midwall FS. Thus, midwall EF can be used to monitor LV systolic dysfunction, which is not possible with conventional LVEF and LVFS.

Midwall FS has been used to detect depressed LV systolic function in patients with LVH [1,3-5]. A previous study found significant differences in midwall measurements of the fiber shortening and lengthening velocities in normal and hypertrophic patients [16]. The midwall FS measurement is preferred because systolic wall thickening is non-uniform, with the inner wall thickening to a substantially greater extent than the outer wall. This may be because fibers in the subendocardial and subepicardial myocardium are orientated longitudinally, whereas those in the midwall region are orientated circumferentially [4,7]. Ishizu et al. also showed differences in radial strain between the inner and outer halves of the myocardium and differences in circumferential strain among the three layers in the endocardial, midwall, and epicardial myocardium by strain analysis using 2D STE [17]. For these reasons, the LV midwall FS is a more physiologically appropriate measurement of LV systolic performance in patients with LVH, compared to conventional FS [1,2,4-9]. However, midwall FS measurements are inherently flawed because of foreshortening errors and reliance upon geometric models that may be inaccurate in the diseased heart [18]. Also, echocardiographic calculation of midwall FS is a geometry-based index derived from linear measurement of the posterior and septal walls, and consequently cannot distinguish between septal and posterior wall function [2]. Therefore, calculation of midwall FS is made from a limited region of the LV. In contrast, midwall EF can estimate foreshortening without use of a geometric model because midwall EF is calculated in planes. Thus, measurement of midwall EF is a relatively new approach that can mitigate the errors inherent in midwall FS.

The correlation of LVMI with midwall EF was higher than with TDI parameters. It has been suggested that TDI can be used to quantify regional ventricular function objectively and the mitral annular velocity may be a more sensitive index of LV function [19]. However, TDI is particularly affected by translational and tethering effects, and angle-dependency. Therefore, there is some limitation in interpretation of measurements by TDI. In contrast, analysis of midwall EF is relatively free of the influence of these adverse affects. Thus, in a clinical setting, measurement of midwall EF is effective for quantifying the impairment of LV function due to LVH. It is reported that the longitudinal strain is a useful method for assessing myocardium systolic dysfunction in patients with LVH [20]. Nonetheless, the correlation of LVMI with midwall EF was higher than with longitudinal strain in our study. Midwall EF measurements may be superior to detect myocardial systolic dysfunction in patients with LVH than longitudinal strain.

Our study also showed the usefulness of 2D STE for measurement of midwall EF. The STE technique relies on tracking of natural acoustic markers in the myocardium from frame to frame throughout the cardiac cycle using a sum of absolute differences algorithm [10]. Thus, the application of 2D STE method has been widely used in the study of subclinical or overt LV dysfunction [21]. Evaluation of midwall EF by 2D STE does not require difficult or lengthy acquisition and offline reconstruction, which are impractical in routine clinical use. The 2D STE method also allows automatic measurements of LV volume to be performed without the need for manual tracings. We positioned the initial ROI manually on the midpoint of the wall thickness at the end of diastole only. Then, we automatically obtained the volume curve using a speckle tracking algorithm throughout the cardiac cycle. In systole, the initial ROI was not positioned at the midpoint of the wall thickness because the systolic thickening of the inner layer is larger than that of the outer layer. Ishizu et al. also proved this phenomenon using a speckle tracking method [17]. Thus, our method is similar to the mathematical midwall mechanism reported in previous studies [1,2,4-9] and may be clinically useful and is likely to have low observer variability.

In this study, midwall EF correlated with the degree of LVH. Patients with LVH have intrinsic dysfunction in both systole and diastole. Our study showed that midwall EF can detect LV systolic dysfunction, which cannot be detected by conventional EF. This may be an important sign of LV dysfunction in patients with hypertension, which may not always be due to diastolic dysfunction, but can often be caused by systolic dysfunction, as assessed by midwall EF [6]. Evaluation of midwall EF may allow assessment of LV systolic performance in patients with various LV geometries. Our method is relatively easy to apply in conventional echocardiography, with clinical settings similar to those for volume measurement by the routine biplane method.

Our methods demonstrated that the midwall EF is clinically useful for detecting the systolic function in addition to midwall FS, TDI, and strain. Midwall EF can detect LV systolic dysfunction, which cannot be detected by conventional EF. The intrinsic systolic dysfunction may affect predictive of subsequent morbidity and mortality in patients with LVH. Midwall EF will have possibilities to detect the beneficial change of intrinsic systolic dysfunction by medical treatment in clinical settings.

## Study limitation

The current study has several limitations. First, we did not examine the influence of afterload, which may affect the midwall analysis. Second, the subjects did not constitute a consecutive series and were selected according to image quality. Finally, midwall EF using 2D STE is not theoretically the same as conventional midwall FS, which is needed to measure both systole and diastole.

## Conclusion

The study showed that midwall EF can be evaluated using 2D STE. The midwall EF in the LVH group was significantly lower than that in the control group and midwall EF was correlated with the degree of LVH. This measurement can be used to detect LV systolic dysfunction, which cannot be detected by conventional EF. Thus, evaluation of midwall EF may allow assessment of new parameters of LV systolic function in patients with various LV geometries.

## Abbreviations

LVH: Left ventricular hypertrophy; FS: Fractional shortening; EF: Ejection fraction; 2D: 2-dimensional; STE: Speckle tracking echocardiography; LVMI: Left ventricular mass index; TDI: Tissue Doppler imaging; IVST: Interventricular septal thickness; PWT: Posterior wall thickness; LVDd: Left ventricular end-diastolic diameter; LVDs: Left ventricular end-systolic diameter; LAD: Left atrial dimension; SV: Stroke volume; DCT: Deceleration time; E velocity: Peak early mitral flow velocity; A velocity: Peak late mitral flow velocity; E/A: Ratio of mitral E and A; S’: The early systolic mitral annular velocity; E’: The early diastolic mitral annular velocity; A’: The late diastolic annular velocity; ROI: Regions of interests.

## Competing interests

The authors have no competing interests.

## Authors’ contributions

HY, MS, and GH planned the study, investigated all patients, performed measurements and analyzed the data. YK and TO analyzed data and wrote the manuscript. MN and KS made critical review of the paper. All authors read and approved the final manuscript.
